# Knowledge and attitudes towards clinical trials among women with ovarian cancer: results of the ACTO study

**DOI:** 10.1186/s13048-022-00970-w

**Published:** 2022-04-14

**Authors:** Paola Mosconi, Anna Roberto, Nicoletta Cerana, Nicoletta Colombo, Florence Didier, Maurizio D’Incalci, Domenica Lorusso, Fedro Alessandro Peccatori, Network of Clinicians and Participants (1), Grazia Artioli, Luigi Cavanna, Rita Ceccherini, Giovanna Cirigliano, Giuseppe Comerci, Gennaro Cormio, Alessandra Crippa, Alberto Farolfi, Antonio Febbraro, Donatella Giardina, Stefano Greggi, Maurizio Lalle, Mariateresa Lapresa, Marina Marzola, Carla Merisio, Anna Maria Mosconi, Michele Peiretti, Giuseppe Ricci, Graziana Ronzino, Giovanni Scambia, Paolo Scollo, Federica Sina, Giulia Carlo Stella, Federica Tomao, Patrizia Vici, Paolo Zola

**Affiliations:** 1 Laboratorio di Ricerca per il coinvolgimento dei cittadini in sanità, Dipartimento di Salute Pubblica, Istituto di Ricerche Farmacologiche Mario Negri IRCCS, Via Mario Negri 2, 20156 Milano, Italy; 2 ACTO Alleanza Contro il Tumore Ovarico Onlus, Milano, Italy; 3 Istituto Europeo di Oncologia IRCCS and University of Milan-Bicocca, Milano, Italy; 4 Istituto Europeo di Oncologia IRCCS, Milano, Italy; 5 Humanitas IRCCS, Rozzano (MI), Italy; 6 Fondazione Policlinico Universitario Agostino Gemelli IRCCS, Roma, Italy

**Keywords:** Ovarian Cancer, Knowledge, Advocacy group, Survey

## Abstract

**Background:**

Despite several initiatives by research groups, regulatory authorities, and scientific associations to engage citizens/patients in clinical research, there are still obstacles to participation. Among the main discouraging aspects are incomplete understanding of the concepts related to a clinical trial, and the scant, sometimes confused, explanations given. This observational, cross-sectional multicenter study investigated knowledge, attitudes and trust in clinical research.

We conducted a survey among women with ovarian cancer at their first follow-up visit or first therapy session, treated in centers belonging to the Mario Negri Gynecologic Oncology (MaNGO) and Multicenter Italian Trials in Ovarian Cancer (MITO) groups. A questionnaire on knowledge, attitudes and experience was assembled ad hoc after a literature review and a validation process involving patients of the Alliance against Ovarian Cancer (ACTO).

**Results:**

From 25 centers 348 questionnaire were collected; 73.5% of responders were 56 years or older, 54.8% had a high level of education, more than 80% had no experience of trial participation. Among participants 59% knew what clinical trials were and 71% what informed consent was. However, more than half did not know the meaning of the term randomization. More than half (56%) were in favor of participating in a clinical trial, but 35% were not certain. Almost all responders acknowledged the doctor’s importance in decision-making. Patients’ associations were recognized as having a powerful role in the design and planning of clinical trials.

**Conclusions:**

This study helps depict the knowledge and attitudes of women with ovarian cancer in relation to clinical trials, suggesting measures aimed at improving trial “culture”, literacy and compliance, and fresh ways of communication between doctors and patients.

**Supplementary Information:**

The online version contains supplementary material available at 10.1186/s13048-022-00970-w.

## Introduction

Advances in medicine and healthcare are based on research, first in laboratories and then through clinical studies. Currently there are several initiatives by research groups, regulatory authorities, patients’ advocacy groups or scientific societies to encourage citizens’ and patients’ participation in the discussion of research priorities and partnership, mainly based on the assumption that where there is clinical research patients’ care is better [1, 2]. Various materials, websites, tutorials and videos, have been developed in different languages for lay people and patients, to explain the fundamental concepts of clinical trials and faster awareness and participation [3–5]. There are also other interesting efforts to promote active involvement and partnership [6, 7].

Nevertheless, among the obstacles that researchers generally encounter in presenting and conducting clinical trials, the involvement and adhesion of patients stand out [8, 9], as well as the scant knowledge citizens and patients have about clinical research [10–12]. Other discouraging aspects are the incomplete understanding of the concepts related to a trial, particularly the randomization methods, and the scarce—sometimes confusing—explanations received from the doctor or healthcare staff presenting it. Finally, there is fear of adverse events given the uncertainty about the new therapy, the possibility of direct or indirect costs or additional medical visits—which may be problematic for the patients and the caregiver—or the feeling of being subject to an experiment [13, 14]. The main reasons for participation include an altruistic feeling of being able to improve health care for other patients and society, access to free, innovative therapies, and being closely monitored [15, 16].

Knowledge, attitudes and experience related to clinical research, particularly randomized controlled clinical trials (RCT), are recognised as the best approach to reach hard, reproducible results, are not widely found among lay people and participants [17, 18], especially ovarian cancer patients, despite fruitful research in this area. Ovarian cancer is the 10th female tumor, and still one of the biggest killers, with high mortality: five-year survival does not exceed 40%, 31% at ten years [19].

The Alliance against Ovarian Cancer (ACTO) is an Italian non-profit organization, with seven regional branches and more than a thousand associates that aims to create a strong alliance between patients, researchers, and doctors to fight ovarian cancer (https://www.acto-italia.org/it). ACTO decided to investigate issues related to trial participation, considering that despite the low incidence of ovarian cancer, clinical research is making constant progress through collaborative research groups.

The aims of this observational, cross-sectional multicenter study are to investigate how familiar clinical research is, particularly RCT, to women with ovarian cancer and how they react to the proposal to participate in a clinical trial. The following areas will be considered: knowledge and understanding, confidence, obstacles and reasons for participating, and satisfaction with the information received. An ad hoc picture of the knowledge, attitudes and experience of these patients with clinical trials will be useful in order to promote trial participation and improve the general perception of research among personally involved women and, consequently, their families and society.

## Material and methods

Italian centers belonging to the MaNGO-Mario Negri Gynecologic Oncology [20] and MITO-Multicenter Italian Trials in Ovarian Cancer [21] groups were invited to participate. Women with a confirmed diagnosis of epithelial ovarian cancer, signing informed consent, were included in the study at the first follow-up visit and/or the first therapy session. Exclusion criteria were women not understanding Italian, those with life expectancy less than six months and women in phase 1 trials. Each participant center was asked to present the study to eligible women, to collect signatures for informed consent, and to deliver the first questionnaire. After three months, the coordinator center mailed the follow-up questionnaire to women willing to participate in an RCT and giving consent to be further contacted in order to find out their direct experience.

The study started in March 2019, and finished in September 2020 after several months of delay due to the SARS-CoV2 pandemic.

### Questionnaire

The questionnaire was developed starting from literature, and through a process involving researchers, clinicians and ACTO regional branches (Puglia, Campania, Lombardia, Piemonte, and Lazio) (Supplementary 1).

#### Item generation

In 2018, a literature search restricted to papers published after 1 January 2000 identified 125 articles, 19 of them eligible according to the protocol, and four others were added from references. As no questionnaire fitted the protocol we collected questions from selected articles and set out a comparative table according to three domains: knowledge, attitudes and experience. Depending on the frequencies and relevance we selected a set of questions and adapted them to the Italian setting, with simple, direct language.

#### Validity of content

A pilot test among ten research experts in the Coordinator Center evaluated the completeness of each domain (irrelevant questions, and aspects not taken into account), and the clarity of the questions. Seven proposed re-formulating some questions, and layout changes. The proposed changes were discussed and implemented, leading to the second version of the two questionnaires.

#### Test (field-test)

New versions were discussed during a meeting of representatives of ACTO branches. Participants received a copy of the questionnaires in advance together with a form to collect comments and feedback on completeness, clarity, timing and difficulty (based on the EORTC Debriefing Form model) [22]. All feedbacks were discussed during the meeting. This led to revision of the questionnaires: two questions were added in the first questionnaire and revisions were made to some questions and answers in both the first and second questionnaires. Finally, the last versions of the questionnaires were sent to a convenience sample of target women identified by ACTO representatives’ group. No significant request emerged, and only one question was modified.

After discussion with the ACTO representatives’ group, we decided to use a paper questionnaire rather than a web tool, considering it the best method to ensure greater adherence to the study, facilitating the participation of women who are not used to technological tools.

### Sample size

In view of descriptive nature of the study, no statistical hypothesis was formulated and no formal calculation of the sample size. A recruitment period of six months was established.

### Statistical analyses

Descriptive analyses were conducted using SAS statistical software, version 9.4, which allows crossing variables according to several criteria. A statistical test (*p*-value) was used only for results showing a significant trend, with alpha 0.05.

## Results

Were collected 359 questionnaires from 25 participant centers; 348 were included in the analysis as 11 had to be excluded: 1 withdrew consent, 3 were collected after the end of the study, and 7 were not from patients with ovarian cancer. Among the 80 eligible responders for the follow up questionnaire, 62 women were reached as they provided a valid address; 17 follow-up questionnaires were collected but their results are not included in this article.

Table 1 lists the responders’ main characteristics. Most were 56 years or older, half had high school education, and most did not have paid work; 56.3% had been diagnosed with ovarian cancer in the previous two years, and more than 80% had not taken part in any clinical trial in the past, but 26% had been invited to enter a clinical trial in the previous three months.

**Table 1:** Main characteristics of 348 responders

	**No. (%) **^**a**^
**Age**	
Less than 55 years	92 (26.5)
56–65 years	124 (35.6)
More than 66 years	132 (37.9)
**Education**	
Elementary or lower middle	157 (45.2)
High school or degree	190 (54.8)
**Employment**	
Paid work (full or part-time)	95 (28.9)
No paid work (retired, housewife, other)	234 (71.1)
**Work in a healthcare profession**	
Yes	21 (6.2)
No	316 (93.8)
**Year of diagnosis of ovarian cancer**	
2019–2020	196 (56.3)
Before 2019	152 (43.7)
**In the past have you ever been invited to take part in a clinical trial?**	
Yes	40 (11.6)
No	305 (88.4)
**In the past three months have you been invited to participate in a randomized clinical trial?**	
Yes	88 (26.0)
No	250 (73.9)

^a^Discrepancies in the total are due to missing values (less than 6%)

The best-known aspect of clinical research was informed consent. About 60% of women had read about clinical trials on the internet, in magazines, or on tv, or had discussed it with their physician, and about 60% knew that ethics committee authorisation was required before starting a trial. Only 34.9% knew the meaning of randomization (Table 2). These percentages were higher in the small sample of 21 women working in health settings. Education and a longer history of illness were associated with more knowledge about clinical research (Supplementary 2).

**Table 2:** Knowledge about clinical trials

	**No. (%) **^**a**^
**Have you ever read on the Internet or in newspapers, heard on television or discussed with your doctor anything about a "clinical study" or "clinical trial"?**	
Yes	202 (59.1)
No	140 (40.9)
**Do you know what the term “randomization” means referring to the investigation of a new drug or medical procedure?**	
Yes	120 (34.9)
No	224 (65.1)
**Have you ever heard about "informed consent" in relation to clinical research?**	
Yes	247 (71.8)
No	97 (28.2)
**Do you know that starting a clinical trial requires the approval of an ethics committee, made up of people with different skills, who assess the scientific validity, quality, and feasibility of the trial?**	
Yes	202 (59.4)
No	138 (40.6)

^a^Discrepancies in the total are due to missing values (less than 3%)

Six aspects were assessed to investigate attitudes toward clinical trials (Table 3). In general responders agreed that clinical trials benefit patients and society (91.5%), and that the doctor has a very important part in the decision (90.3%). Half the responders said “I don’t know” about the risk/benefit ratio, but would agree to participate themselves or encourage a relative or friend. Responders acknowledged that all the results, positive or negative, were to be published in scientific articles and lay publications. The level of education particularly influenced the question about on publishing all the data: 71.9% vs 85.5% in the more educated sample, while the duration of illness influenced all the responses, also lowering the rates of "I don't know".

**Table 3:** Attitudes towards clinical trials

	**No. (%) **^**a**^
**Clinical trials benefit patients and society**	
Strongly agree/Agree	202 (91.5)
I don’t know	27 (8.2)
Strongly disagree/ Disagree	1 (0.3)
**The risks of participating in a clinical trial outweighs the potential benefits**	
Strongly agree/Agree	36 (11.4)
I don’t know	171 (54.1)
Strongly disagree/ Disagree	109 (34.5)
**The doctor is important in the decision to participate in a clinical trial**	
Strongly agree/Agree	290 (90.3)
I don’t know	23 (7.2)
Strongly disagree/ Disagree	8 (2.5)
**If asked, I would be in favor of participating in a clinical trial**	
Strongly agree/Agree	180 (56.8)
I don’t know	118 (35.2)
Strongly disagree/ Disagree	19 (6.0)
Missing	31
**I would also encourage a relative or friend to participate in a clinical trial**	
Strongly agree/Agree	176 (55.4)
I don’t know	118 (37.1)
Strongly disagree/ Disagree	24 (7.5)
**All clinical trial results, positive or negative, must be made public in scientific articles and lay publications**	
Strongly agree/Agree	257 (77.9)
I don’t know	55 (16.7)
Strongly disagree/ Disagree	18 (5.4)

^a^Discrepancies in the total are due to missing values (than 10%)

Full disclosure of the advantages and disadvantages with a clearly defined reference group of healthcare professionals was the most important aspect for participation in a clinical trial. Also relevant are the usefulness of the data for future patients, and a clear description of duties. Participants were particularly interested in the purposes of data collection and storage (Table 4).

**Table 4:** Clinical trials and participation

	**No. (%)**
**Select the three answers you consider most important before taking part in a clinical trial**	
Full information on the advantages and disadvantages	296 (30.2)
Physicians or health professionals for reference	234 (23.8)
Confidence that the results will be useful for future patients	193 (19.7)
A clear description of how it will be conducted and what participation implies (visits, extra costs, etc.)	190 (19.4)
Information material to consult independently	39 (3.9)
Insurance coverage	14 (1.4)
Who finances the study (non-profit organizations or associations, pharmaceutical companies, private companies, etc.)	15 (1.5)
**For greater security in the use of personal data collected during a clinical trial, you need to know … (select 2 answers)**	
For what purpose the data is collected	221 (34.9)
By whom, where, and for how long it will be stored	134 (21.2)
Who has access to the data	94 (14.8)
How participants' privacy will be ensured	88 (13.9)
Consent will be required to use the data in other studies	56 (8.8)
How to modify or withdraw consent to use of the data at any time	40 (6.3)

Most responders (91.5%) thought it right that doctors—when they have already data in favor of a new treatment but not certain compared to what is already available—ask patients to participate in a clinical trial. Responders thought that the good of the patient and the community (47.2% of preferences), together with progress in science and medicine (42.9% of preferences), were more important for a doctor to invite a patient to take part in a trial than difficulties in treating the patient (5.6%) or personal gain (2.4%) or pharmaceutical company interests (1.8%).

Regarding patients’ associations, 71.2% of responders agreed that their involvement in design and planning is important, recognising a role in providing information and facilitating participation more than discussing the trial plan or the results (Table 5).

**Table 5:** Involvement of patients’ associations in designing and planning clinical trials

	**No. (%)**
**Should the representatives of citizens and patients be actively involved in the design and planning of a clinical trial?**	
Yes	237 (71.2)
No	96 (28.8)
Missing	15
**If Yes, what role have representatives of citizens and patients (select 2 answers)**	
Improve the information given to patients about the trial	96 (20.9)
Facilitate patients’ participation in the trial	93 (20.3)
Make suggestions for clinical trials of real benefit to patients	91 (19.8)
Help with financing the trial	56 (12.2)
Discuss the clinical trial plan to make it better	55 (11.9)
Communicate the trial results	39 (8.5)
Act as the spokesman for patients during analysis and discussion of the results	29 (6.3)

Finally, 80 (90.9%) of the 88 women invited to participate in a clinical trial during this study agreed. Among their reasons they cited confidence in the doctor (32% of preferences), benefits to society (22.9%), access to new therapies that are not otherwise available (21.7%), and because the clinical trial offers the best possible treatment (10.2%).

## Discussion

This study gives a snapshot of the knowledge and attitudes of women with a history of ovarian cancer on clinical trials. Most of the participants knew what clinical trials and informed consent are but more than half did not know what the term randomization meant, and were unable to evaluate the risk–benefit ratio of participation. Half the participants were in favor of participating and about a third were not certain. In case of clinical uncertainty about the best treatment, doctors have a right to ask a patient to participate in a clinical trial and are—as almost all responders said—important in the decision-making. Patients’ associations are recognized as having a powerful role in the design and planning of clinical trials. Only a small percentage of women in this study had been asked to participate in a trial in the past, even among those with a longer history of the disease. This is in line with a survey by the World Ovarian Cancer Coalition which reported 12% of participation in clinical trials [10]. However, in our sample only eight of the 88 women invited to participate in a trial in the past three months had refused.

In accordance with the mission of ACTO, these results should lead to important advocacy to promote more research in this specific area of gynaecologic oncology and to invite more women to participate in clinical research. Population-wide action to raise the awareness of clinical trials is also needed, possibly jointly among cancer patient organizations. The levels of knowledge about clinical research and trials reflect the need for greater attention to health literacy and empowerment [23–26]. The promotion of clinical research and trials among healthy people, before they become patients, is also needed to facilitate participation in clinical research and decisions [14, 27].

In general there is a positive attitude towards clinical research and trials, recognizing the benefits for patients and society; however, responders expressed uncertainty when assessing the balance between risk and benefits. Personal decisions to participate- themselves or relatives or friends- showed a high level of indecision. In line with other experiences, these results suggest the need to boost confidence in clinical research, and the greater involvement of clinicians when a trial is proposed to patients [28, 29]. The majority in our sample thought doctors have an important role in a decision, and confidence in the doctor is the first reason to agree to participate.

Uncertainty about the results of any experimental treatments, and the fear of failure or death, might explain both hesitancy and a passive role in decisions. It is well known that a cancer diagnosis and the related complex decisions, such as participation in a trial, can create emotional stress or anxiety [30]. The disease is often associated with uncertainty and fear of death and the request to participate in a trial may increase anxiety; anxiety and depression are both associated with hesitancy in making decisions [31]. Furthermore, anxiety is linked to greater engagement in threat-avoidance behaviors, and depression is linked to lower engagement in reward-seeking behaviors [32]. Emotions are potent, pervasive, predictable, sometimes harmful and sometimes beneficial drivers of decisions. Across different domains, important regularities appear in the mechanisms through which emotions influence judgments and choices [33, 34]. So increased awareness of emotions may help putting them to best use and reducing their influence as a bias in shared decisions [35].

Our study confirms that there is still ample room to improve and implement shared decision-making in oncology. In a review Covvey et al. [36] identified barriers to shared decision-making: uncertainty in the treatment decision, concern about adverse effects, and poor physician communication. They describe themes for facilitators for shared decision-making including the physician’s consideration of the patient’s preferences, the physician’s positive actions and behavior, and the use or encouragement of support systems. As our study shows, the patient-physician relationship can influence patients' preferences for and processing information. An informed decision can be facilitated by considering each individual patient's knowledge, values, and emotional and cognitive decisional skills. Taking account of all these factors can therefore help improve shared decision-making, possibly increasing patients’ participation in clinical trials.

The role of associations is widely recognised, in line with a general consensus in the literature and among cooperative groups about partnership in clinical research discussions and projects [37]. Partnership with patients’ representatives may mitigate the difficulties due to poor retention rates, impact, low level and clinical significance of the study [38]. Patient associations, besides promoting detailed scientific information and offering psychological support, foster clinical research based on patients' needs, helping develop feasible, good-quality clinical trials [39, 40].

This study has some limitations. First of all, the representativeness of the sample collected: the participating centers are all centers of excellence, specialized in ovarian cancer treatment and participating in clinical research through national or international multicenter trials. This may have boosted the women’s confidence in the study, and in the physicians involved. Secondly, not all the centers invited participated and the numbers of patients involved by each center ranged from 1 to 45, and there is no information about patients who refused to participate. While it is true that the Covid-19 pandemic influenced the accrual of patients, it is also true that this type of study -academic, cross-sectional, observational- tends to be less attractive to clinicians than interventional trials. Thirdly, the preparation of local documents for the Ethics Committee influenced the participation of several centers. On average, 181 days were required for approval, with a range of 66–362 days, thus further reflecting the difficulties in coordinating this study. Finally, the data collected with the second questionnaire was too limited for any analysis.

In conclusion, knowledge and attitudes towards participation in clinical research have important implications for their success. This study adds useful information to a larger project aimed at improving the culture of clinical trials and larger-scale awareness. As regards shared decision-making, Covvey et al [36] showed that the most common cancers studies are breast and prostate, but the strength of this study is that it provides information on ovarian cancer the top five causes of cancer deaths among women between the ages of 50 and 69 years.

A new decision-making process about participation in a trial and the involvement of healthcare professionals to back up the physicians—including research nurses, case managers and psychologists—should be examined for an engagement model fostering clinical research. Correct information, especially for less educated women with a shorter history of disease, must be carefully considered. Shared decision-making facilitates patient-centered care and is increasingly important in oncology, where patients are faced with multifaceted treatment decisions that require them to weigh efficacy and safety, quality of life, and cost. It takes time and effort for physicians and patients to communicate straightforwardly and they still face communication barriers. The shared decision-making with ovarian cancer patients has to be developed, while concentrating on understanding a patient’s fears, emotions and reactions better. Exploring patients’ psychological needs could help physicians boost their engagement in clinical research, and dedicated healthcare professionals would be particularly useful when patients experience high levels of distress, which can create difficulties in decision-making about trial participation.

Patients’ associations, besides providing support and comfort by giving a sense of belonging and through mutual help, are important partners in clinical research, providing scientific information, promoting the culture of partnership and supporting the active participation of patients in decisions. The results of this study could be helpful for advocate groups and clinicians to implement concrete actions for raising awareness on the importance of participation in clinical research.

## Supplementary Information

**Additional file 1:** **Supplementary 1. **The ACTO questionnaire.

**Additional file 2:** **Supplementary 2.** Knowledge about clinical trials according to education and history of ovarian cancer (% of Yes).

## Data Availability

The data analysed for this study are available from the corresponding author on reasonable request.

## Abbreviations

ACTO: Alleanza contro il tumore ovarico Onlus, Alliance against ovarian cancer
MaNGO: Mario Negri Gynecologic Oncology
MITO: Multicenter Italians Trials in Ovarian Cancer
RCT: Randomized controlled trials
