# Supplementary material for: Knowledge and attitudes towards clinical trials among women with ovarian cancer: results of the ACTO study
Source: J Ovarian Res. 2022 Apr 14;15:45. doi: 10.1186/s13048-022-00970-w (PMC9010065; doi:10.1186/s13048-022-00970-w)
Supplement: Supplementary file 1 — Additional file 1: Supplementary 1. The ACTO questionnaire. [file 13048_2022_970_MOESM1_ESM.docx]

***Ricerca in-ACTO Q1***

Dear Madam,

Thank you for taking part in this project to collect knowledge and opinions on clinical research and participation in clinical trials. Please answer all the questions: there is no right or wrong answer- just choose the answer you feel is best for you.

After you have completed the questionnaire you can hand it at the ward, send it using the pre-stamped envelope provided, or scan it and e-mail it to: ricercainacto@marionegri.it

Thanks for your help

Mario Negri Institute IRCCS & ACTO Alliance Against Ovarian Cancer

**LET’ START WITH SOME QUESTIONS ABOUT YOU**

| **Age** | ⚫ less than 50 years \|__\|  ⚫ 50-55 years \|__\|  ⚫ 56-60 years \|__\|  ⚫ 61-65 years \|__\|  ⚫ more than 65 years \|__\| |
| --- | --- |
| **Education** | ⚫ Elementary \|__\|  ⚫ Lower middle school \|__\|  ⚫ High school \|__\|  ⚫ Degree or more \|__\| |
| **Employment** | ⚫ Paid work (full or part-time) \|__\|  ⚫ No paid work (retired, housewife, other) \|__\| |
| **Do you work in a healthcare profession (doctor, nurse ...)?** | Yes \|__\| No \|__\| |
| **When did you receive the diagnosis of ovarian cancer?** | /__/__/day /__/__/month /__/__/__/__/ year |
| **In the past, have you ever been invited to take part in a clinical trial?** | Yes \|__\| No \|__\| |

**WE ARE NOW INTERESTED IN FINDING OUT HOW FAMILIAR YOU ARE**

**WITH SOME ASPECTS OF CLINICAL RESEARCH**

**Have you ever read on the Internet or in newspapers, heard on television or discussed with your doctor anything about a "clinical study" or "clinical trial"?**

Yes |__| No |__|

**Do you know what the term “randomization” means referring to the investigation of a new drug or medical procedure?**

Yes |__| No |__|

**Have you ever heard about "informed consent" in relation to clinical research?**

Yes |__| No |__|

**Do you know that starting a clinical trial requires the approval of an Ethics Committee, made up of people with different skills, who assess the scientific validity, quality, and feasibility of the trial?**

Yes |__| No |__|

**In your opinion, in the next 15 years what impact will science and technological innovations have on medical care and health?**

Negative impact |__| No impact |__| Positive impact |__| I don’t know |__|

**WHAT IS A RANDOMIZED CLINICAL TRIAL ?**

When you want to know if a new treatment is safe and works better than what is already available, doctors and researchers organize a clinical trial, as the best way to get an answer. A clinical trial, thanks to volunteers who sign a consent form, compares different treatments randomly assigned to patients - the so-called "randomization" - to make up comparable groups.

During a clinical trial, doctors use the same treatment procedures, collect data and follow up patients for a pre-set period. The results are therefore as reliable as possible.

**Please indicate the three answers you consider most important before taking part in a clinical trial like the one outlined here.**

□ Full information on the advantages and disadvantages

□ A clear description of how it will be conducted and what participation implies (visits, extra costs, etc.)

□ Confidence that the results will be useful for future patients

□ Information material to consult independently

□ Have a group of physicians or health professionals for reference

□ Be covered by insurance

□ Know who finances the study (non-profit organizations or associations, pharmaceutical companies, private companies, etc.)

**Do you think it right that doctors, when they have data in favor of a new treatment compared to one already available, but not certain - ask patients to participate in a clinical trial?**

Yes |__| No |__|

**In your opinion, why does a doctor invite you to take part in a clinical trial?**

**Please indicate the two answers you consider most important**

□ For the good of the patient and community

□ Because otherwise he would not know how to treat the patient

□ For personal gain

□ To foster the progress of science and medicine

□ For a pharmaceutical company’s interests

**Please state your degree of agreement or disagreement with the following statements ...**

|  | *Strongly disagree* | *Disagree* | *I don’t*  *know* | *Agree* | *Strongly agree* |
| --- | --- | --- | --- | --- | --- |
| Clinical trials benefit patients and society | 1 | 2 | 3 | 4 | 5 |
| The risks of participating in a clinical trial outweigh the potential benefits | 1 | 2 | 3 | 4 | 5 |
| The doctor plays an important role in the decision to participate in the study | 1 | 2 | 3 | 4 | 5 |
| If asked, I would be in favor of participating in a clinical trial | 1 | 2 | 3 | 4 | 5 |
| I would also encourage the participation of a relative or friend in a clinical trial | 1 | 2 | 3 | 4 | 5 |
| All clinical trial results, positive or negative, must be made public in scientific articles and lay publications | 1 | 2 | 3 | 4 | 5 |

**In your opinion, should representatives of citizens and patients be actively involved during design the planning of a clinical trial?**

Yes |__| No |__|

**If YES, what could they do?**

**Please indicate the two answers you consider most important**

□ Make suggestions for conducting clinical trials of real benefit to patients

□ Discuss the clinical trial plan to make it better

□ Facilitate patients’ participation in the trial

□ Help with financing the trial

□ Improve the information given to patients about the trial

□ Act as spokesman for patients during analysis and discussion of the results

□ Communicate the results of the clinical trial

**For greater security in the use of personal data collected during a clinical trial, you need to know ... please indicate the two answers you consider most important.**

□ By whom, where, and for how long it will be stored

□ For what purpose the data is collected

□ How to modify or withdraw consent to use of the data at any time

□ How participants' privacy will be ensured

□ Who has access to the data

□ Consent will be required to use the data in other studies

**In the past three months, have you been invited to participate in any randomized clinical trial like the one outlined on page 2?**

**No** |__| 🢣 The questionnaire is finished, thanks

**Yes** |__| 🢣 Please answer the last questions

**Have you decided to participate in the randomized clinical trial proposed to you?**

**NO**

**What are the reasons that prompted you NOT TO PARTICIPATE?**

**Please indicate the two answers you consider most important**

□ The information regarding the trial was unclear and inadequate

□ There may be additional costs for visits and/or transport that I cannot afford

□ For personal reasons (for example, I cannot guarantee that I can follow the schedule of visits and/or cycles of therapy set down in the clinical trial, etc.)

□ I generally have little confidence in clinical research in Italy

□ My family and / or friends advised me against it

□ I trust my doctor and I prefer him to select my therapy

□ The possibility of not receiving the new, experimental treatment scares me

□ I didn't have enough time to choose

**YES**

**What are the reasons that led you to PARTICIPATE?**

**Please indicate the two answers you consider most important**

□ Access to new therapies that are not otherwise available

□ Clinical trials are beneficial to society, so I can make a contribution

□ The clinical trial offers me the best possible treatment

□ I have a lot of confidence in the doctor who proposed the trial to me and rely on him

□ My family and friends recommended it

□ The completeness and clarity of the information received

□ I didn't feel I could refuse

□ I can get more assistance and more frequent checkups

Did the information you received at the beginning of the trial seem clear and complete, so you could make an informed and aware choice?

Yes |__| No |__|

Did you have a chance to ask the doctor questions to clarify doubts or curiosity?

Yes |__| No |__|

If Yes, were you satisfied with the answers?

Yes |__| No |__|
