# Supplementary material for: Knowledge and attitudes towards clinical trials among women with ovarian cancer: results of the ACTO study
Source: J Ovarian Res. 2022 Apr 14;15:45. doi: 10.1186/s13048-022-00970-w (PMC9010065; doi:10.1186/s13048-022-00970-w)
Supplement: Supplementary file 2 — Additional file 2: Supplementary 2. Knowledge about clinical trials according to education and history of ovarian cancer (% of Yes). [file 13048_2022_970_MOESM2_ESM.docx]

**Supplementary 2 - Knowledge about clinical trials according to education and history of ovarian cancer (% of Yes)**

**
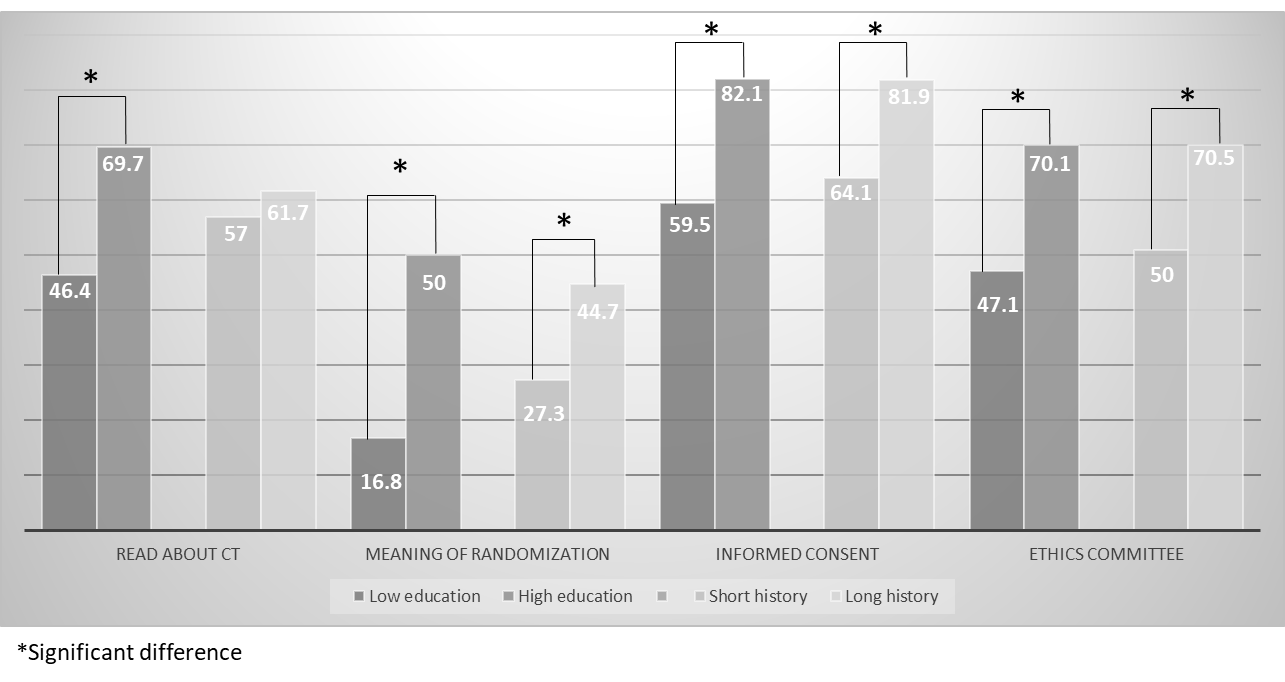
**
